# Psychological impact of mass quarantine on population during pandemics—The COVID-19 Lock-Down (COLD) study

**DOI:** 10.1371/journal.pone.0240501

**Published:** 2020-10-22

**Authors:** Deeksha Pandey, Suvrati Bansal, Shubham Goyal, Akanksha Garg, Nikita Sethi, Dan Isaac Pothiyill, Edavana Santhosh Sreelakshmi, Mehmood Gulab Sayyad, Rishi Sethi

**Affiliations:** 1 KMC Manipal, Manipal Academy of Higher Education (MAHE), Manipal, India; 2 Ashoka University, Sonipat, India; 3 Abeda Inamdar College, Pune, India; 4 King George’s Medical University (KGMU), Lucknow, India; University of Sao Paulo Medical School, BRAZIL

## Abstract

**Background:**

Quarantine often is an unpleasant experience. The aim of this study is to explore the degree of psychological distress in terms of–Depression, Anxiety and Stress among the adult population in India during the strict 21 days mandatory lockdown. We hypothesize that quantification of psychological impact of current situation will help us to modify the policies and implementation strategies. This assessment might also help in future to keep targeted services in place, to cope up with the psychological distress of the quarantined population.

**Method:**

A cross sectional survey design was adopted to assess the psychological state of general population in India, during the COVID-19 mandatory lockdown period, with the help of a validated questionnaire.

**Findings:**

The reported prevalence of depression was around 30.5%, which was the highest among the variables of psychological health. Anxiety was reported by 22.4%, followed by stress which was seen in 10.8% of respondents. In the third week the incidence of depression (37.8% versus 23.4%; p<0.001), anxiety (26.6% versus 18.2%; p<0.001) and stress (12.2% versus 9.3%; p<0.045) was reported to be significantly higher as compared to second week.

**Interpretation:**

Our results suggest a progressively detrimental impact of lockdown on various aspects of psychological health. We noticed around eight to ten fold increase in the prevalence of depression (30.5%) and anxiety (22.4%) during lockdown, as compared to baseline statistics in Indian population (3·1–3·6% for depressive disorders and 3·0–3·5% for anxiety disorders).

## Introduction

The entire world is facing a crisis today with the pandemic associated with the coronavirus disease (COVID-19). Unprecedented policies and strategies are being implement to contain the spread of this disease, that have resulted in around a third of global population being subjected to COVID-19 lockdown. In scientific terminology the word ‘Lockdown’ means ‘Restrictive Mass Quarantine’. Historically in 2003 citywide quarantines were imposed in areas of China and Canada during the outbreak of severe acute respiratory syndrome (SARS). Entire villages in many west African countries were quarantined during the 2014 Ebola outbreak [1]. However, current COVID-19 seems to be the largest and most restrictive quarantine till date.

Quarantine often is an unpleasant experience. On one hand for those who earn their living on daily wages, it is a question of their survival. On the other hand, the loss of freedom, separation from the loved ones, and the uncertainty over the disease status, may pose immense psychological turbulence, even in the more affluent population. These unnatural circumstances have been hypothesized to lead to extreme risk-taking behaviours–including suicidal tendencies. Social isolation and loneliness are recognised risk factors for suicidal attempts [2]. It is imperative to carefully weigh and strategically implement the potential benefits of mandatory mass quarantine against the possible odds of psychological distress in the society [3]. A recent textual analysis of 5780 publications enforces the need of global research collaborations, during this pandemic, in order to address the knowledge gaps in a country to country based approach [4].

The aim of this study is to explore the degree of psychological distress in terms of–Depression, Anxiety and Stress among the adult population in India during the strict 21 days mandatory lockdown. We hypothesize that quantification of psychological impact of current situation will help us to modify the policies and implementation strategies. This assessment might also help in future to keep targeted services in place, to cope up with the psychological distress of the quarantined population.

## Material and methods

### Ethical considerations

The study was conducted in accordance with the Declaration of Helsinki. An expedited Institutional Ethical approval from Kasturba Hospital Manipal, Manipal Academy of Higher Education (MAHE), India was obtained (IEC 248/2020).

### Setting & participants

A cross sectional survey design was adopted to assess the psychological state of general population in India, during the COVID-19 mandatory lockdown period, with the help of a validated questionnaire. Government of India declared a three week complete lockdown starting on 24^th^ of March 2020. Conceptualization and detailed planning was done; ethical clearance was sough in the first week. Data collection took place in week two and three of lockdown.

The inclusion criteria included individuals who were Indian residents above 18 years of age, were literate and had access to our recruiting platforms, that comprised–social networking forums, email services and various messenger groups. Individuals who were not well versed with the English language, did not having access to internet, or were not willing to participate were excluded.

To obtain maximum number of responses in the limited time interval, a snowball sampling strategy was utilized to disseminate the online survey. All respondents provided online informed written consent to participate.

### Survey instrument

We used the international validated and widely used Depression, Anxiety, Stress Scale (DASS-21), as the clinical assessment tool to measure psychological distress. It contains 21 items divided into three subscales of depression, anxiety, and stress, with 7 items allocated for each subscale. The items are scored on a 4-point scale ranging from 0 (did not apply to me at all) to 3 (applied to me very much, or most of the time). The range of score a participant could get for each subscale varied from 0 to 21. The recommended cut-off points were used to classify participants into normal, mild, moderate, severe, and extremely severe in terms of depression, anxiety, and stress.

We also collected demographic details (age, gender, marital status, education and occupation) of the participants incorporated in the same online survey.

### Statistical analysis

The data on categorical variables is shown as n (% of respondents—prevalence) and the data on continuous variables is presented as mean and standard deviation (SD). The inter-group statistical comparison of distribution of categorical variables is tested using Pearson’s Chi-Square test. In the entire study, p-values less than 0.05 are considered to be statistically significant. The entire data is statistically analyzed using Statistical Package for Social Sciences (SPSS ver 22.0, IBM Corporation, USA) for MS Windows.

## Results

We received responses from 1395 in the study duration of two weeks. The distribution of responses received each day was almost uniform.

### Details of demography

Mean age of the cohort of respondents was 25.0 ± 10.2 years and the minimum–maximum age range was 18–73 years. Most of our respondents (82.8%) belonged to less than 30 years with 50.4% in the age bracket of 18–20 years. Most of them (82.7%) were unmarried and were students (76%). The number of women respondents (58.1%) was slightly more than the men. Of 1395 respondents participated in the study, 582 (41.7%) were male, 805 (57.7%) were female and 8 (0.6%) respondents had marked the ‘other’ category and were excluded from the statistical analysis on gender Table 1.

**Table 1:** Distribution of demographic parameters of the respondents participated in the study (n = 1395).

Parameter		No. of respondents	% of respondents
Age group	≤20 years	703	50.4
	21–30 years	452	32.4
	31–40 years	77	5.5
	41–50 years	101	7.2
	>50 years	62	4.4
Gender	Male	582	41.9
	Female	805	58.1
Educational status	Primary / Secondary	439	31.5
	Graduate	633	45.4
	Post-Graduate/Doctoral	323	23.1
Marital status	Unmarried	1153	82.7
	Married	234	16.8
	Divorced	8	0.5
Occupational status	Student	1060	76.0
	Private service	82	5.9
	Govt. service	48	3.4
	Self employed	158	11.3
	Other	47	3.4

### Overall psychological health

The reported prevalence of depression was around 30.5%, which was the highest among the variables of psychological health. Anxiety was reported by 22.4%, followed by stress which was seen in 10.8% of respondents. Stress was of only mild to moderate degree. However a few reported severe degree of depression (1.7%) and anxiety (2.2). Ten respondents (0.7%) even reported having extremely severe level of anxiety Table 2.

**Table 2:** Overall distribution of level of depression, anxiety and stress among the respondents participated in the study (n = 1395).

		No. of respondents	% of respondents
Depression	Normal	969	69.5
	Mild	194	13.9
	Moderate	208	14.9
	Severe	24	1.7
Anxiety	Normal	1083	77.6
	Mild	113	8.1
	Moderate	159	11.4
	Severe	30	2.2
	Extremely severe	10	0.7
Stress	Normal	1245	89.2
	Mild	117	8.4
	Moderate	33	2.4

### Impact of duration of lockdown on psychological health

**Fig 1:**
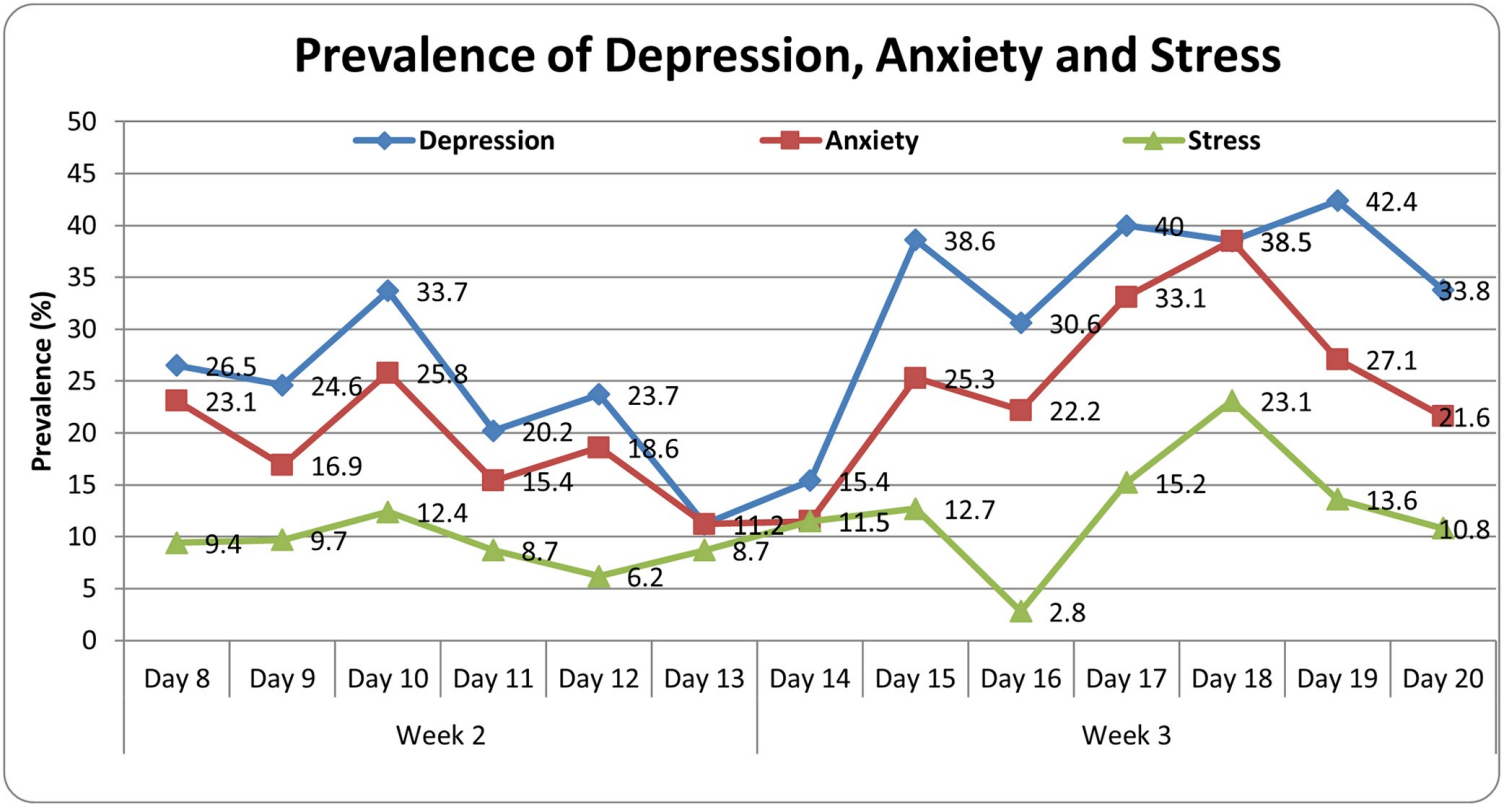
Impact of duration of lockdown on psychological health. This graph depicts day wise prevalence of depression, anxiety and stress in our study cohort.

On week-wise analysis of impact of lockdown we found an increasing negative impact of lockdown. The survey was conducted during second and third week of lockdown. In the third week the incidence of depression (37.8% versus 23.4%; p<0.001), anxiety (26.6% versus 18.2%; p<0.001) and stress (12.2% versus 9.3%; p<0.045) was reported to be significantly higher as compared to second week. In the scale of severity also all the studied components of mental health showed an deteriorating trend Table 3. Day wise prevalence of depression, anxiety and stress is depicted in Fig 1.

**Table 3:** Distribution of level of depression, anxiety and stress according to duration of lockdown among the respondents participated in the study (n = 1395).

		Duration of Lockdown				
		Week 2 (n = 708)		Week 3 (n = 687)		P-value
		n	%	n	%	
Depression	Normal	542	76.6	427	62.2	0.001***
	Mild	87	12.3	107	15.6	
	Moderate	69	9.7	139	20.2	
	Severe	10	1.4	14	2.0	
Anxiety	Normal	579	81.8	504	73.4	0.001***
	Mild	55	7.8	58	8.4	
	Moderate	54	7.6	105	15.3	
	Severe	11	1.6	19	2.8	
	Extremely severe	9	1.3	1	0.1	
Stress	Normal	642	90.7	603	87.8	0.045*
	Mild	47	6.6	70	10.2	
	Moderate	19	2.7	14	2.0	

P-value by Chi-Square test, P-value<0.05 is considered to be statistically significant.

*P-value<0.05,

***P-value<0.001.

### Factors affecting psychological health

We noticed that distribution of level of depression, anxiety and stress, varied significantly across various age groups of respondents (P<0.001). Significantly higher proportion of respondents with younger age had mild to severe level of depression compared to the relatively older respondents (P<0.001). Significantly higher proportion of women had mild to severe level of depression (P<0.002), anxiety (P<0.002) and stress (P<0.001) as compared to the men. Lower educational level was associated with higher prevalence of psychological disorders (P<0.001). Significantly higher proportion of unmarried respondents had mild to severe level of depression, anxiety and stress, as compared to married or divorced group respondents in the study (P<0.001).

## Discussion

Our results suggest a progressively detrimental impact of lockdown on various aspects of psychological health. We noticed around eight to ten fold increase in the prevalence of depression (30.5%) and anxiety (22.4%) during lockdown, as compared to baseline statistics in Indian population (3·1–3·6% for depressive disorders and 3·0–3·5% for anxiety disorders) [5].

Concerns related to morbidity and mortality of disease during pandemic can have enormous impact of psychological wellbeing of individuals. Imposed quarantine resulting in a loss of control and a sense of being trapped, may intensify and exacerbate it.

In December 2019, a cluster of acute respiratory tract infection cases were identified in Wuhan, China. The cause was a newly identified β-coronavirus. The world health organisation (WHO) officially named the disease as coronavirus disease 2019 (COVID- 19). Soon evidence showed its sustained human-to-human transmission, that became the cause of concern as well as panic. The unique characteristic of COVID -19 is that it is highly contagious though maybe less virulence [6, 7]. Thus the focal point of present strategy to prevent its spread is social isolation. In that attempt several countries including India have gone into a state of complete lockdown. Following up the trend of increasing number of new cases and related deaths as well as looking at the projected models, the future appears uncertain. Researchers and policy makers thus are emphasizing the need of strengthening or supplementing the current measures of containment so as to flatten the curve of spread and to gain time during which a definite treatment or vaccine may be developed [8, 9].

However, the uncertain epidemiological benefits of this new form of mandatory mass quarantine over the uncertain psychological costs, needs to be ascertained carefully and in an evidence based manner [3]. Mass quarantine, due to a pandemic is likely to increase depression, anxiety, stress levels substantially. The sudden psychological turmoil may also have a knock-on effect on other physical health issues [10].

Similar to our results a study among the Weibo users in china during the beginning of COVID-19 epidemic found an increase in negative emotions (anxiety, depression, and indignation), as well as a decrease in positive emotions (Oxford happiness) and life satisfaction. They suggested using social media data for timely understanding of the impact of public health emergencies on the public’s mental health during the epidemic period. However invasion of the privacy may be an ethical concern here [11].

Another recent study used social media platform for exploring the impact of COVID. They collected all related tweets using the Twitter Standard Search Application Programming Interface (API). This study however did not focus in the analysis of psychological behaviour aspect [12].

In the month of January 2020, within three weeks into the epidemic, Chinese Government had imposed travel restrictions and lockdown affecting more than 50 million people in total. The first psychological survey in the general population in China within the first two weeks of the COVID-19 outbreak, noted that 53.8% respondents rated psychological impact of outbreak as moderate or severe. It was an online survey wherein responses from 1210 respondents were analysed [13]. They found that 16.5% of respondents reported moderate to severe depressive symptoms and 8.1% reported moderate to severe level of stress. The prevalence of anxiety was comparatively more (28.8%) in their cohort. Whereas in our study population depression was the most frequently reported symptom. This may be dependent on the time span of study. Initially the unknown aetiology, untraced exposure and exponential expansion of numbers would have been the cause of anxiety. Soon the cause was identified, pathophysiology and transmission understood, yet the inability to combat the pandemic effectively would have made the general public more depressed about the whole situation. Imposed social isolations and various restriction could have played a role in intensified depression.

A nationwide Chinese survey and a longitudinal study conducted in China, both revealed that during mass quarantine, with the passage of time, distress levels among the public showed a descending trend [14, 15]. Quite contrary to their results we found a deterioration of psychological health in our study cohort, with the passage of time. They have attributed the improvement to effective prevention and control measures adopted by the Chinese Government. A very important step taken by National Health Commission of China (NHC) in this regard was to integrate psychological crisis intervention into the general deployment of disease prevention. This was specially done to reduce the risk of negative psychological outcomes caused by the COVID-19 outbreak and promote social stability [16, 17]. Though the government of India is implementing novel strategies to combat the crisis related to physical health, evidence-based evaluations of psychological impact and mental health interventions are relatively scarce.

We found that the most affected group in our study was the young adults in the age group of 18–30 years, who were still pursuing their education. Other studies have also reported this group to be the most vulnerable psychologically during such lockdown situations [13–15, 18, 19]. It is hypothesized to be related to the tech friendly nature of young adult generation today. This makes them more prone to get affected and stressed because of access to unrestricted but non-validated information available on internet and social media platforms.

With the free availability and access to internet and social media platforms there is significant concerns about the real versus fake news [3, 20]. Experience from Korea and Vietnam emphasises the need of a public system to verify the validity of information available [21, 22]. It is imperative that while authorities focus on physical health, there must be consistent efforts to enhance social support systems, cater emotional needs and eliminate stigma of the disease [23]. Though combating the physical disease is the priority during a pandemic, strategies to address mental health issues in such challenging times should not be ignored [14].

Women were more susceptible to suffer from all forms of psychological symptoms (depression, anxiety and stress) as compared to men; which is similar to earlier studies [13–15].

Most of the studies on the psychological impact of the current pandemic and lockdown have come from China, because of the obvious reason of the province being the first one to get effected. A somewhat similar study from Spain among 1310 subjects observed high degree of distress and loneliness among young adults, women and those who had negative self- perception of aging [24].

This kind of situation, during the mass quarantine for COVID-19 has definitely increases the possibility of psychological problems. Quarantine gradually distances people from each other against their will and in the absence of interpersonal communication, depression and anxiety are more likely to occur and worsen. On the other hand face to face psychological counselling and consultation is difficult. There is an urgent need to brainstorm and develop novel strategies to lessen the burden of psychological issues in a quarantined society. Telephonic conversations or internet based counselling from a reliable source can be helpful. Structured letter therapy has been suggested as a kind of feasible psychological intervention approach during times of mass quarantine for COVID-19 in China [25]. In this time of crisis, one of the strategies for the practitioners may be to promptly start adopting e-mental health care applications. This would help in two ways–in providing continued care to current patients in need and it will also help as a new strategy to cope with the imminent upsurge in psychological symptoms due to the pandemic and resulting lockdown [26]. In today’s world of social media, we should try to use this power effectively for positive mass education and motivation. In this era of mass internet supported communication, this present crisis is the first ever global crisis of this magnitude [27]. We need to turn to the telepsychiatry experts to formulate best processes to address the urgent psychological need of the society [28]. Online cognitive behaviour therapy and mindfulness based therapy will be helpful for patients [29]. Varies psychotherapies to deal with specific psychological issues have been discussed in a recently published review targeting the current pandemic [30]. Implementation of Online mental health services have their own challenges in Asian Countries like India and China [31]. Policies and strategies need to be specific and targeted to the population under focus. Thus more studies and data is required from masses.

Our study is the first one from the Indian subcontinent to focus on the emerging psychological distress during times of mass quarantine. It also tries to identify the high risk population in a relatively large cohort of participants across India. The limitation however is our sampling method which might be biased towards the group of subjects who are more tech savvy. Thus, it might not be a true reflection of entire population in India. However, as our cohort and sampling has been very random across various states of the country, occupations, educational status and age groups, the finding are generalizable to the literate strata of the Indian society.

## Conclusion

The COVID-19 pandemic and lockdown situation seems to have ignited another pandemic of depression, anxiety and stress. The psychological impact of a mandatory mass quarantine should be weighed more thoughtful and in an evidence based manner. In order to combat present crisis of increasing mental distress among population, novel psychological intervention strategies that are feasible and accessible are the need of the hour.

## Supporting information

S1 File (DOCX)
