# Supplementary material for: Psychological impact of mass quarantine on population during pandemics—The COVID-19 Lock-Down (COLD) study
Source: PLoS One. 2020 Oct 22;15(10):e0240501. doi: 10.1371/journal.pone.0240501 (PMC7580982; doi:10.1371/journal.pone.0240501)
Supplement: S1 File — (DOCX) [file pone.0240501.s001.docx]

**S1 Table. Distribution of level of depression, anxiety and stress among the respondents participated in the study (n=1395).**

|  |  | **No. of respondents** | **% of respondents** |
| --- | --- | --- | --- |
| Depression | Normal | 969 | 69.5 |
|  | Mild | 194 | 13.9 |
|  | Moderate | 208 | 14.9 |
|  | Severe | 24 | 1.7 |
| Anxiety | Normal | 1083 | 77.6 |
|  | Mild | 113 | 8.1 |
|  | Moderate | 159 | 11.4 |
|  | Severe | 30 | 2.2 |
|  | Extremely severe | 10 | 0.7 |
| Stress | Normal | 1245 | 89.2 |
|  | Mild | 117 | 8.4 |
|  | Moderate | 33 | 2.4 |

***Distribution of level of depression***

Of 1395 respondents participated in the study, 969 (69.5%) had no depression, 194 (13.9%) had mild depression, 208 (14.9%) had moderate depression and 24 (1.7%) had severe depression.

***Distribution of level of anxiety***

Of 1395 respondents participated in the study, 1083 (77.6%) had no anxiety, 113 (8.1%) had mild anxiety, 159 (11.4%) had moderate anxiety, 30 (2.2%) had severe anxiety and 10 (0.7%) had extremely severe anxiety.

***Distribution of level of stress***

Of 1395 respondents participated in the study, 1245 (89.2%) had no stress, 117 (8.4%) had mild stress, 33 (2.4%) had moderate stress.

**S2 Table. Distribution of level of depression, anxiety and stress according to age group of the respondents participated in the study (n=1395).**

|  |  | **Age Group (years)** | | | | | | | | | |  |
| --- | --- | --- | --- | --- | --- | --- | --- | --- | --- | --- | --- | --- |
|  |  | **≤20 (n=703)** | | **21 – 30 (n=452)** | | **31 – 40 (n=77)** | | **41 – 50 (n=101)** | | **>50 (n=62)** | | **P-value** |
|  |  | **n** | **%** | **n** | **%** | **n** | **%** | **n** | **%** | **n** | **%** |  |
| Depression | Normal | 441 | 62.7 | 321 | 71.0 | 66 | 85.7 | 95 | 94.1 | 46 | 74.2 | 0.001^***^ |
|  | Mild | 105 | 14.9 | 68 | 15.0 | 9 | 11.7 | 5 | 5.0 | 7 | 11.3 |  |
|  | Moderate | 143 | 20.3 | 61 | 13.5 | 1 | 1.3 | 1 | 1.0 | 2 | 3.2 |  |
|  | Severe | 14 | 2.0 | 2 | 0.4 | 1 | 1.3 | 0 | 0.0 | 7 | 11.3 |  |
| Anxiety | Normal | 524 | 74.5 | 349 | 77.2 | 68 | 88.3 | 93 | 92.1 | 49 | 79.0 | 0.001^***^ |
|  | Mild | 67 | 9.5 | 37 | 8.2 | 1 | 1.3 | 6 | 5.9 | 2 | 3.2 |  |
|  | Moderate | 95 | 13.5 | 52 | 11.5 | 7 | 9.1 | 2 | 2.0 | 3 | 4.8 |  |
|  | Severe | 17 | 2.4 | 12 | 2.7 | 1 | 1.3 | 0 | 0.0 | 0 | 0.0 |  |
|  | Extremely severe | 0 | 0.0 | 2 | 0.4 | 0 | 0.0 | 0 | 0.0 | 8 | 12.9 |  |
| Stress | Normal | 620 | 88.2 | 401 | 88.7 | 71 | 92.2 | 100 | 99.0 | 53 | 85.5 | 0.001^***^ |
|  | Mild | 71 | 10.1 | 40 | 8.8 | 5 | 6.5 | 0 | 0.0 | 1 | 1.6 |  |
|  | Moderate | 12 | 1.7 | 11 | 2.4 | 1 | 1.3 | 1 | 1.0 | 8 | 12.9 |  |
| P-value by Chi-Square test, P-value<0.05 is considered to be statistically significant. ***P-value<0.001. | | | | | | | | | | | | |

***Distribution of level of depression according to age***

Distribution of level of depression differs significantly across various age groups of respondents participated in the study (P-value<0.001). Significantly higher proportion of respondents with younger age had mild to severe level of depression compared to the relatively older respondents participated in the study (P-value<0.001).

***Distribution of level of anxiety according to age***

Distribution of level of anxiety differs significantly across various age groups of respondents participated in the study (P-value<0.001). Significantly higher proportion of respondents with younger age had mild to severe level of anxiety compared to the relatively older respondents participated in the study (P-value<0.001).

***Distribution of level of stress according to age***

Distribution of level of stress differs significantly across various age groups of respondents participated in the study (P-value<0.001). Significantly higher proportion of respondents with younger age had mild to moderate level of stress compared to the relatively older respondents participated in the study (P-value<0.001).

**S3 Table. Distribution of level of depression, anxiety and stress according to gender of the respondents participated in the study (n=1387^§^).**

|  |  | **Gender** | | | |  |
| --- | --- | --- | --- | --- | --- | --- |
|  |  | **Male (n=582)** | | **Female (n=805)** | | **P-value** |
|  |  | **n** | **%** | **n** | **%** |  |
| Depression | Normal | 438 | 75.3 | 531 | 66.0 | 0.002^**^ |
|  | Mild | 72 | 12.4 | 122 | 15.2 |  |
|  | Moderate | 66 | 11.3 | 141 | 17.5 |  |
|  | Severe | 6 | 1.0 | 11 | 1.4 |  |
| Anxiety | Normal | 483 | 83.0 | 600 | 74.5 | 0.001^***^ |
|  | Mild | 48 | 8.2 | 65 | 8.1 |  |
|  | Moderate | 45 | 7.7 | 114 | 14.2 |  |
|  | Severe | 6 | 1.0 | 24 | 3.0 |  |
|  | Extremely severe | 0 | 0.0 | 2 | 0.2 |  |
| Stress | Normal | 542 | 93.1 | 703 | 87.3 | 0.002^**^ |
|  | Mild | 34 | 5.8 | 83 | 10.3 |  |
|  | Moderate | 6 | 1.0 | 19 | 2.4 |  |
| P-value by Chi-Square test, P-value<0.05 is considered to be statistically significant. **P-value<0.01, ***P-value<0.001. **^§^**Of 1395 total respondents, a total of 8 responses were excluded from the statistical analysis for gender characteristic due to invalid selection of category. | | | | | | |

***Distribution of level of depression according to gender***

Distribution of level of depression differs significantly between group of male and group of female respondents participated in the study (P-value<0.01). Significantly higher proportion of female respondents had mild to severe level of depression compared to male respondents participated in the study (P-value<0.01).

***Distribution of level of anxiety according to gender***

Distribution of level of anxiety differs significantly between group of male and group of female respondents participated in the study (P-value<0.001). Significantly higher proportion of female respondents had mild to extremely severe level of anxiety compared to male respondents participated in the study (P-value<0.001).

***Distribution of level of stress according to gender***

Distribution of level of stress differs significantly between group of male and group of female respondents participated in the study (P-value<0.01). Significantly higher proportion of female respondents had mild to moderate level of stress compared to male respondents participated in the study (P-value<0.01).

**S4 Table. Distribution of level of depression, anxiety and stress according to educational status of the respondents participated in the study (n=1395).**

|  |  | **Educational Status** | | | | | |  |
| --- | --- | --- | --- | --- | --- | --- | --- | --- |
|  |  | **Primary / Secondary (n=439)** | | **Graduate (n=633)** | | **Post-Graduate/Doctoral (n=323)** | | **P-value** |
|  |  | **n** | **%** | **n** | **%** | **n** | **%** |  |
| Depression | Normal | 262 | 59.7 | 435 | 68.7 | 272 | 84.2 | 0.001^***^ |
|  | Mild | 73 | 16.6 | 89 | 14.1 | 32 | 9.9 |  |
|  | Moderate | 98 | 22.3 | 99 | 15.6 | 11 | 3.4 |  |
|  | Severe | 6 | 1.4 | 10 | 1.6 | 8 | 2.5 |  |
| Anxiety | Normal | 319 | 72.7 | 485 | 76.6 | 279 | 86.4 | 0.001^***^ |
|  | Mild | 45 | 10.3 | 53 | 8.4 | 15 | 4.6 |  |
|  | Moderate | 62 | 14.1 | 77 | 12.2 | 20 | 6.2 |  |
|  | Severe | 12 | 2.7 | 17 | 2.7 | 1 | 0.3 |  |
|  | Extremely severe | 1 | 0.2 | 1 | 0.2 | 8 | 2.5 |  |
| Stress | Normal | 383 | 87.2 | 556 | 87.8 | 306 | 94.7 | 0.001^***^ |
|  | Mild | 45 | 10.3 | 65 | 10.3 | 7 | 2.2 |  |
|  | Moderate | 11 | 2.5 | 12 | 1.9 | 10 | 3.1 |  |
| P-value by Chi-Square test, P-value<0.05 is considered to be statistically significant. ***P-value<0.001. | | | | | | | | |

***Distribution of level of depression according to educational status***

Distribution of level of depression differs significantly across various educational groups of respondents participated in the study (P-value<0.001). Significantly higher proportion of respondents with lower educational status had mild to severe level of depression compared to relatively highly educated respondents participated in the study (P-value<0.001).

***Distribution of level of anxiety according to educational status***

Distribution of level of anxiety differs significantly across various educational groups of respondents participated in the study (P-value<0.001). Significantly higher proportion of respondents with lower educational status had mild to extremely severe level of anxiety compared to relatively highly educated respondents participated in the study (P-value<0.001).

***Distribution of level of stress according to educational status***

Distribution of level of stress differs significantly across various educational groups of respondents participated in the study (P-value<0.001). Significantly higher proportion of respondents with lower educational status had mild to moderate level of stress compared to relatively highly educated respondents participated in the study (P-value<0.001).

**S5 Table. Distribution of level of depression, anxiety and stress according to marital status of the respondents participated in the study (n=1395).**

|  |  | **Marital Status** | | | | | |  |
| --- | --- | --- | --- | --- | --- | --- | --- | --- |
|  |  | **Unmarried (n=1153)** | | **Married (n=234)** | | **Divorced (n=8)** | | **P-value** |
|  |  | **n** | **%** | **n** | **%** | **n** | **%** |  |
| Depression | Normal | 753 | 65.3 | 210 | 89.7 | 6 | 75.0 | 0.001^***^ |
|  | Mild | 172 | 14.9 | 20 | 8.5 | 2 | 25.0 |  |
|  | Moderate | 205 | 17.8 | 3 | 1.3 | 0 | 0.0 |  |
|  | Severe | 23 | 2.0 | 1 | 0.4 | 0 | 0.0 |  |
| Anxiety | Normal | 862 | 74.8 | 214 | 91.5 | 7 | 87.5 | 0.001^***^ |
|  | Mild | 103 | 8.9 | 10 | 4.3 | 0 | 0.0 |  |
|  | Moderate | 149 | 12.9 | 9 | 3.8 | 1 | 12.5 |  |
|  | Severe | 29 | 2.5 | 1 | 0.4 | 0 | 0.0 |  |
|  | Extremely severe | 10 | 0.9 | 0 | 0.0 | 0 | 0.0 |  |
| Stress | Normal | 1011 | 87.7 | 227 | 97.0 | 7 | 87.5 | 0.001^***^ |
|  | Mild | 111 | 9.6 | 5 | 2.1 | 1 | 12.5 |  |
|  | Moderate | 31 | 2.7 | 2 | 0.9 | 0 | 0.0 |  |
| P-value by Chi-Square test, P-value<0.05 is considered to be statistically significant. ***P-value<0.001. | | | | | | | | |

***Distribution of level of depression according to marital status***

Distribution of level of depression differs significantly across various marital status group of respondents participated in the study (P-value<0.001). Significantly higher proportion of unmarried respondents had mild to severe level of depression compared to married or divorced group respondents participated in the study (P-value<0.001).

***Distribution of level of anxiety according to marital status***

Distribution of level of anxiety differs significantly across various marital status group of respondents participated in the study (P-value<0.001). Significantly higher proportion of unmarried respondents had mild to extremely severe level of anxiety compared to married or divorced group respondents participated in the study (P-value<0.001).

***Distribution of level of stress according to marital status***

Distribution of level of stress differs significantly across various marital status group of respondents participated in the study (P-value<0.001). Significantly higher proportion of unmarried respondents had mild to moderate level of stress compared to married or divorced group respondents participated in the study (P-value<0.001).

**S6 Table. Distribution of level of depression, anxiety and stress according to occupational status of the respondents participated in the study (n=1395).**

|  |  | **Occupational status** | | | | | | | | | |  |
| --- | --- | --- | --- | --- | --- | --- | --- | --- | --- | --- | --- | --- |
|  |  | **Student (n=1060)** | | **Private Service (n=82)** | | **Govt. Service (n=48)** | | **Self employed (n=158)** | | **Other (n=47)** | | **P-value** |
|  |  | **n** | **%** | **n** | **%** | **n** | **%** | **n** | **%** | **n** | **%** |  |
| Depression | Normal | 685 | 64.6 | 74 | 90.2 | 43 | 89.6 | 131 | 82.9 | 36 | 76.6 | 0.001^***^ |
|  | Mild | 158 | 14.9 | 4 | 4.9 | 5 | 10.4 | 24 | 15.2 | 3 | 6.4 |  |
|  | Moderate | 201 | 19.0 | 3 | 3.7 | 0 | 0.0 | 3 | 1.9 | 1 | 2.1 |  |
|  | Severe | 16 | 1.5 | 1 | 1.2 | 0 | 0.0 | 0 | 0.0 | 7 | 14.9 |  |
| Anxiety | Normal | 794 | 74.9 | 75 | 91.5 | 40 | 83.3 | 139 | 88.0 | 35 | 74.5 | 0.001^***^ |
|  | Mild | 92 | 8.7 | 4 | 4.9 | 5 | 10.4 | 8 | 5.1 | 4 | 8.5 |  |
|  | Moderate | 143 | 13.5 | 2 | 2.4 | 3 | 6.3 | 11 | 7.0 | 0 | 0.0 |  |
|  | Severe | 29 | 2.7 | 1 | 1.2 | 0 | 0.0 | 0 | 0.0 | 0 | 0.0 |  |
|  | Extremely severe | 2 | 0.2 | 0 | 0.0 | 0 | 0.0 | 0 | 0.0 | 8 | 17.0 |  |
| Stress | Normal | 930 | 87.7 | 80 | 97.6 | 47 | 97.9 | 149 | 94.3 | 39 | 83.0 | 0.001^***^ |
|  | Mild | 107 | 10.1 | 0 | 0.0 | 1 | 2.1 | 9 | 5.7 | 0 | 0.0 |  |
|  | Moderate | 23 | 2.2 | 2 | 2.4 | 0 | 0.0 | 0 | 0.0 | 8 | 17.0 |  |
| P-value by Chi-Square test, P-value<0.05 is considered to be statistically significant. ***P-value<0.001. ‘Other’ category includes – retired, homemakers and other unclassified occupations. | | | | | | | | | | | | |

***Distribution of level of depression according to occupational status***

Distribution of level of depression differs significantly across various occupational groups of respondents participated in the study (P-value<0.001). Significantly higher proportion of student respondents had mild to severe level of depression compared to group of respondents with other occupational status participated in the study (P-value<0.001).

***Distribution of level of anxiety according to occupational status***

Distribution of level of anxiety differs significantly across various occupational groups of respondents participated in the study (P-value<0.001). Significantly higher proportion of student respondents had mild to extremely severe level of anxiety compared to group of respondents with other occupational status participated in the study (P-value<0.001).

***Distribution of level of stress according to occupational status***

Distribution of level of stress differs significantly across various occupational groups of respondents participated in the study (P-value<0.001). Significantly higher proportion of student respondents had mild to moderate level of stress compared to group of respondents with other occupational status participated in the study (P-value<0.001).

**S7 Table. Distribution of level of depression, anxiety and stress according to duration of lockdown among the respondents participated in the study (n=1395).**

|  |  | **Duration of Lockdown** | | | |  |
| --- | --- | --- | --- | --- | --- | --- |
|  |  | **Week 2 (n=708)** | | **Week 3 (n=687)** | | **P-value** |
|  |  | **n** | **%** | **n** | **%** |  |
| Depression | Normal | 542 | 76.6 | 427 | 62.2 | 0.001^***^ |
|  | Mild | 87 | 12.3 | 107 | 15.6 |  |
|  | Moderate | 69 | 9.7 | 139 | 20.2 |  |
|  | Severe | 10 | 1.4 | 14 | 2.0 |  |
| Anxiety | Normal | 579 | 81.8 | 504 | 73.4 | 0.001^***^ |
|  | Mild | 55 | 7.8 | 58 | 8.4 |  |
|  | Moderate | 54 | 7.6 | 105 | 15.3 |  |
|  | Severe | 11 | 1.6 | 19 | 2.8 |  |
|  | Extremely severe | 9 | 1.3 | 1 | 0.1 |  |
| Stress | Normal | 642 | 90.7 | 603 | 87.8 | 0.045^*^ |
|  | Mild | 47 | 6.6 | 70 | 10.2 |  |
|  | Moderate | 19 | 2.7 | 14 | 2.0 |  |
| P-value by Chi-Square test, P-value<0.05 is considered to be statistically significant. *P-value<0.05, ***P-value<0.001. | | | | | | |

***Distribution of level of depression according to duration of lockdown***

Distribution of level of depression differs significantly between week 2 and week 3 of lockdown period among the respondents participated in the study (P-value<0.001). Significantly higher proportion of respondents had mild to severe level of depression in the third week of lockdown compared to second week of lockdown among the respondents participated in the study (P-value<0.001).

***Distribution of level of anxiety according to duration of lockdown***

Distribution of level of anxiety differs significantly between week 2 and week 3 of lockdown period among the respondents participated in the study (P-value<0.001). Significantly higher proportion of respondents had mild to extremely severe level of anxiety in the third week of lockdown compared to second week of lockdown among the respondents participated in the study (P-value<0.001).

***Distribution of level of stress according to duration of lockdown***

Distribution of level of stress differs significantly between week 2 and week 3 of lockdown period among the respondents participated in the study (P-value<0.05). Significantly higher proportion of respondents had mild to moderate level of stress in the third week of lockdown compared to second week of lockdown among the respondents participated in the study (P-value<0.05).

**S8 Table. Distribution of demographic parameters of the respondents participated in the study (n=1395).**

| **Parameter** |  | **No. of respondents** | **% of respondents** |
| --- | --- | --- | --- |
| Age group | ≤20 years | 703 | 50.4 |
|  | 21 – 30 years | 452 | 32.4 |
|  | 31 – 40 years | 77 | 5.5 |
|  | 41 – 50 years | 101 | 7.2 |
|  | >50 years | 62 | 4.4 |
| Gender | Male | 582 | 41.9 |
|  | Female | 805 | 58.1 |
| Educational status | Primary / Secondary | 439 | 31.5 |
|  | Graduate | 633 | 45.4 |
|  | Post-Graduate/Doctoral | 323 | 23.1 |
| Marital status | Unmarried | 1153 | 82.7 |
|  | Married | 234 | 16.8 |
|  | Divorced | 8 | 0.5 |
| Occupational status | Student | 1060 | 76.0 |
|  | Private service | 82 | 5.9 |
|  | Govt. service | 48 | 3.4 |
|  | Self employed | 158 | 11.3 |
|  | Other | 47 | 3.4 |

***Age Distribution***

Of 1395 respondents participated in the study, 703 (50.4%) had age below 20 years, 452 (32.4%) had age between 21 – 30 years, 77 (5.5%) had age between 31 – 40 years, 101 (7.2%) had age between 41 – 50 years and 62 (4.4%) had age above 50 years. Mean ± SD of age of the whole group of respondents was 25.0 ± 10.2 years and the minimum – maximum age range was 18 – 73 years.

***Gender Distribution***

Of 1395 respondents participated in the study, 582 (41.7%) were male, 805 (57.7%) were female and 8 (0.6%) respondents had marked the ‘other’ category and were excluded from the statistical analysis on gender.

***Distribution of educational status***

Of 1395 respondents participated in the study, 439 (31.5%) had education upto Primary / Secondary, 633 (45.4%) had education upto graduation and 323 (23.2%) had Post-Graduate/Doctoral education.

***Distribution of marital status***

Of 1395 respondents participated in the study, 1153 (82.7%) were unmarried, 234 (16.8%) were married and 8 (0.5%) were divorced.

***Distribution of occupational status***

Of 1395 respondents participated in the study, 1060 (76.0%) were students, 82 (5.9%) had private service, 48 (3.4%) had Govt. service, 158 (11.3%) were self employed and 47 (3.4%) had other occupation.

**S9 Fig. Distribution of level of depression according to various demographic parameters of respondents participated in the study (n=1395).**

**S10 Fig. Distribution of level of anxiety according to various demographic parameters of respondents participated in the study (n=1395).**

**S11 Fig. Distribution of level of stress according to various demographic parameters of respondents participated in the study (n=1395).**
